# PRELIMINARY FINDINGS OF THE SHARING CHOICES INTERVENTION ON PRIMARY OUTCOME MEASURES

**DOI:** 10.1093/geroni/igad104.1526

**Published:** 2023-12-21

**Authors:** Jennifer Wolff, Talan Zhang, Erin Giovannetti, Sri Rebala, David Roth

**Affiliations:** Johns Hopkins University, Baltimore, Maryland, United States; Johns Hopkins School of Medicine, Baltimore, Maryland, United States; MedStar Health Research Institute, Baltimore, Maryland, United States; MedStar Health Research Institute, Baltimore, Maryland, United States; Johns Hopkins University, Baltimore, Maryland, United States

## Abstract

A cluster randomization approach was applied to 55 primary care practices across two health systems. A 1:2 randomization design resulted in 19 practices being randomized to the SHARING Choices intervention condition and 36 practices randomized to a usual care control condition. The primary outcome variables include documentation of an advance directive (AD) or medical orders for life-sustaining treatment in the electronic health record (EHR) within 12 months of exposure to the intervention for all patients and receipt of potentially burdensome care within 6 months of death for patients who die with a serious illness diagnosis. At the time of this abstract submission, preliminary data on AD documentation in the EHR have been analyzed for one of the two health systems. In that health system, among 8,350 patients in intervention practices, AD documentation in the EHR increased from 19.2% to 25.1% one year later, compared to increases from 15.7% to 17.9% among 19,179 patients in control practices. This markedly greater increase for patients in intervention practices was highly significant statistically in a multilevel logistic regression model that took patient clustering within practices into account (adjusted odds ratio = 2.25; 95% confidence interval = 2.01, 2.51; p < 0.001). The SHARING Choices intervention, therefore, more than doubled the odds of having an AD added to the EHR within one year. Preliminary findings on this outcome from the other health system and other outcomes from both health systems will be analyzed in the months ahead and presented at the GSA meeting in November.

